# Association Between Self-Reported Food Preferences and Psychological Well-Being During Perimenopausal Period Among Chinese Women

**DOI:** 10.3389/fpsyg.2020.01196

**Published:** 2020-06-03

**Authors:** Tingting Wu, Xiaorong Hou, Fan Zhang, Manoj Sharma, Yong Zhao, Zumin Shi

**Affiliations:** ^1^School of Public Health and Health Management, Chongqing Medical University, Chongqing, China; ^2^Research Center for Medicine and Social Development, Chongqing Medical University, Chongqing, China; ^3^Collaborative Innovation Center of Social Risks Governance in Health, Chongqing Medical University, Chongqing, China; ^4^College of Medical Informatics, Chongqing Medical University, Chongqing, China; ^5^Department of Behavioral and Environmental Health, Jackson State University, Jackson, MS, United States; ^6^Chongqing Key Laboratory of Child Nutrition and Health, Chongqing, China; ^7^Human Nutrition Department, College of Health Sciences, QU Health, Qatar University, Doha, Qatar

**Keywords:** food preferences, psychological, well-being, mental health, perimenopausal

## Abstract

**Objective:**

The purpose of this study was sought to assess the association between food preferences and Psychological well-being (PWB) in Chinese women undergoing perimenopause and whether the association is different between rural and urban areas.

**Methods:**

This is a longitudinal study of 929 women in perimenopausal period participating in the China Health and Nutrition Survey (CHNS) during 2009 and 2011. Preference for five kinds of food were assessed in face-to-face interviews and the PWB was measured by scoring three self-reported questions with a total score of 15. Multilevel mixed-effects linear regressions were used to estimate the longitudinal association between food preference and PWB scores. In fully adjusted models, dislike for fruits and like for sweetened beverages had regression coefficient (95% CI) for the PWB score of −1.26, (−2.21–0.321) and 0.66 (0.20–1.11), respectively. The above associations were only found among participants in urban areas, with corresponding regression coefficients of −2.61(95% CI = −4.83, −0.39) for dislike fruit and 1.02(95% CI = 0.09, 1.95) for like sweetened beverages.

**Conclusion:**

In conclusion, PWB score was negatively associated with the dislike for fruit but positively associated with the preference for sweetened beverages, especially among participants from urban areas. The longitudinal data indicate that the PWB score of perimenopausal women might be improved by increasing the intake of fruit. Given the adverse effects of sweetened beverages, more research was need between PWB and the sweetened beverages.

## Introduction

Psychological well-being (PWB) is an essential and integral component of health, which is related to cognitive function, social relationships and health status and is a combination of feeling good and effective functioning (Huppert, 2009). The rapidly aging population, urbanization and the growing mental health issues may threaten overall health. The Global Burden of Disease 2013 study estimated that a third of global burden of mental health diseases were found in China and India (Charlson et al., 2016). The “Comprehensive Mental Health Action Plan for 2013–2020” was established by the World Health Assembly in 2013. All member-states of the World Health Organization (WHO) would take actions to improve mental health and to get the attainment of global targets (World Health Organization [WHO], 2018). In China, the government has also established many plans, including emphasizing the importance of mental health issues; the to help individuals with mental problems and support the general population with needs (Zhao et al., 2017).

The perimenopause is defined by the WHO as the period that commences when the first features of approaching menopause begin until at least one year after the final menstrual period, which as the 2–8 years preceding menopause and the one year after final menses (World Health Organization [WHO], 1996). A series of research have identified that perimenopause has long been associated with psychological distress, both anecdotally and clinically; and the risk of this period for both first-episode depression and for depression reoccurrence was increased (Cohen et al., 2006; Fugate Woods, 2006). As an important turning point of profound physical and emotional changes in a woman’s life, women would suffer from autonomic nervous dysfunction and may often feel agitated and irritable due to changes in endocrine function (World Health Organization [WHO], 1996). Peri-menopausal women are prone to unique psychiatric syndromes, such as perimenopausal depression, perimenopausal neurosis, etc (Tarlatzis and Zepiridis, 2003; Gibbs et al., 2015). A study in China found that 88.7% of women during the perimenopausal had psychological symptoms such as fear, worry, irritability, and insomnia, and only 27.3% who experienced psychological symptoms sought for medical help (Xia, 2014). And the prevalence of menopausal syndrome in women aged 45–55 years was generally more than 80% in Chinese women (Sheng-Lian and Rui-Hua, 2010). It is very common in China that most women in perimenopausal period still have to bear the pressure of the family economy. As a culture, they also were concerned about their children’s work, marriage, and family life. In addition, caring for parents, dealing with disharmony between husband and an accumulation of daily problems faced by perimenopausal women are a mix of unavoidable life events. These factors are such as to increase the risk of anxiety and depression. It has been found that high-risk factors leading to depression have a significant association with the menopausal transition (Soares et al., 2005). Some perimenopause women were unable to adapt to the psychological problems caused by physiological changes due to lack health care knowledge, which seriously affects their physical and mental health (Zan, 2016). Keeping health and preventing from illness before menopause is becoming a critical health issue with the aging of population and a steep rise in medical fee.

Previous studies indicate that food may relieve uncomfortable memories and increase happiness among consumers, and emotion is strongly associated with food choices (Sapranaviciute-Zabazlajeva et al., 2017; von Essen and Mårtensson, 2017; Zhao et al., 2017). In fact, the relationship between dietary options and mental health is complex: poor food eating habits may be a trigger for depression, while poor mental health may in turn lead to unhealthy eating habits (Rahe et al., 2015). A study in China found that students who preference for sweet had a higher stress score than those who did not, and students who with high-stress consumed sweets and spicy foods more frequently than those in other groups (Mu Chunying and Zheng, 2014). Further researches with the complexity of the relationship between dietary and mental health are needed.

The linkage between food intake and dietary choices and PWB are of global importance. The food preference may capture respondents’ long-term dietary behaviors. The previous studies have pointed out that higher consumption of fresh vegetables and fruits associated with better PWB, and which could increase happiness, life satisfaction (Mujcic and Oswald, 2016; Sapranaviciute-Zabazlajeva et al., 2017). Studies have shown strong associations between food preferences and PWB in Western countries (Nguyen et al., 2017; Sapranaviciute-Zabazlajeva et al., 2017; Boehm et al., 2018). China has faced rapid social transformation, urbanization, changes in dietary, which had influenced the food consumption over the last two decades (Wu et al., 2017). Fast food restaurants have proliferated and with more processed and convenient food. The number of fast food restaurants increased from 929,125 to 1,981,019 between 2004 and 2012 (Wang et al., 2016). Per capita consumption in China of meats, eggs, and aquatic products increased, respectively, from 20, 5, and 7 kilograms in 1981 to 29, 10, and 12 kilograms in 2004, whereas the per capita grain consumption in China declined from 145 kilograms in 1981 to 78 kilograms in 2004 in urban areas (Dong and Frank, 2015). Accordingly, research on food preferences should be strengthened with rapid change of dietary. Additionally, only one cross-sectional study examined the relationship between psychological well-being and food preference among adults aged over 45 years (Lee et al., 2018).

Unhealthy dietary intake may intensify perimenopausal symptoms (Joanna Sadowska, 2014). The diet could represent one of the most readily modifiable factors as taking into account the risk factors for chronic disease and risk reductions can be observed by simply altering intake or amounts of various food (Lange-Collett, 2002). Accordingly, healthy diet becomes crucial when a woman to transition into perimenopause. This study aimed to assess the association between food preferences and PWB in Chinese women undergoing perimenopause and whether the association is different between rural and urban areas.

## Materials and Methods

### Design and Sample

The data was from the CHNS with multistage and random cluster procedures in nine provinces across China including Liaoning, Shandong, Henan, Jiangsu, Hubei, Hunan, Guangxi, Guizhou, and Heilongjiang. More details of CHNS sampling and cohort profile information have been described in previous researches (Popkin et al., 2010; Zhang et al., 2014). The survey was approved by the institutional review committees of the University of North Carolina (United States) and the National Institute of Nutrition and Food Safety (China). Informed consent was obtained from all participants. Food preference was assessed in 2004, 2006, 2009, 2011, and 2015 surveys, and PWB measurements were conducted among respondents above the age of 45 in the 2006, 2009, and 2011 surveys, but were conducted among those only above the age of 50 in 2015, the data of 2015 were excluded.

**FIGURE 1:**
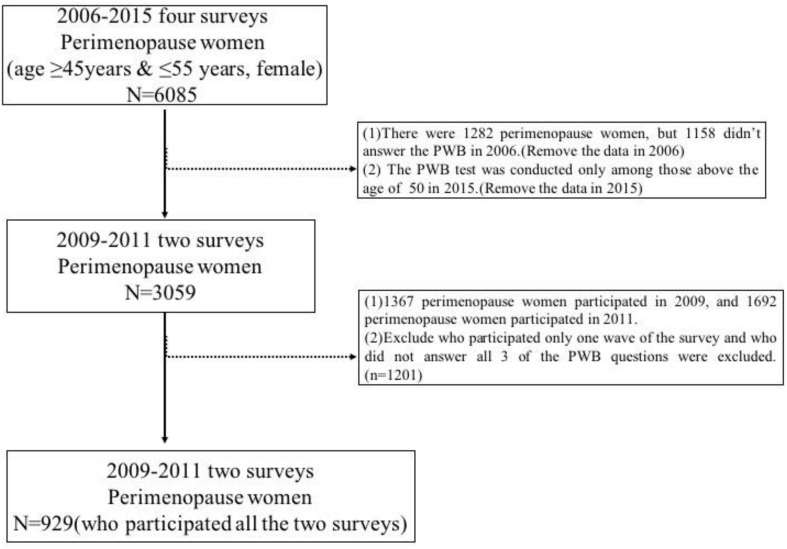
The flowchart of participants attending the CHNS.

This research focused on middle-aged women (45–55) approaching or in perimenopause. The age of 45 to 55 of women, which period was definition of WHO as the natural menopausal time of women and may be a high-risk time for menopausal symptoms (World Health Organization [WHO], 1996). As shown in Figure 1, there were total 6085 women during or approaching perimenopause period from 2006 to 2015 in the CHNS survey with *n* = 1282 in 2006, *n* = 1367 in 2009, and *n* = 1692 in 2011. However, there were 1158 women during or approaching perimenopause period didn’t answer the PWB in 2006. The PWB test was conducted only among those above the age of 50 in 2015. There were 3059 women in perimenopause period attended the PWB test between 2009 and 2011. Participants who only participated one wave of the survey and who did not answer all 3 of the PWB questions were excluded. There were 929 participants participating both years.

### Outcome Variable: PWB Scores

The PWB was measured by asking participants to answer three questions: “I have as much energy as I had in last year”; “I am as happy now as when I was younger”; “As I get older, things are better than I thought they would be.”

These measurements of PWB were specifically designed for those above the age of 45, and all descriptions of questions were translated from Mandarin Chinese to English by the CHNS investigators (Zhang et al., 2014). Participants agreed or disagreed to a certain PWB question was rated on a scale of 1–5, with 1 indicating strong disagreement with question and 5 indicating strong agreement. Next, PWB scores of participants are determined by adding all the three scores. A high score represents a better PWB. We exclude the participants that did not answer all 3 of the PWB questions. The PWB was estimated using data of 2011 and food preferences based on data of 2009.

### Exposure Variable: Food Preference

Food preference was set as the primary predictor based on five food classification questions which include fast food (e.g., pizza and hamburgers), salty snacks (e.g., pretzels and potato chips), fruits, vegetables, and sweetened beverages (including both fruit drinks and soft drinks). Participants were asked “How much do you like this kind of food? (1) Like very much; (2) like; (3) neutral; (4) dislike; (5) dislike it very much (6) don’t eat this food.” Those who reported “like very much” and “like” were defined as “like,” those who reported “dislike” “dislike very much” and “don’t eat this food” were defined as “dislike.”

### Covariates

The basic socioeconomic status variables and disease status were employed as covariates. Age, educational level, employment status, marital status and area and per capita annual family income were described as individuals’ basic socioeconomic status information. Age was treated as a continuous variable, and all other variables were treated as categorical variables. Education was categorized into three-levels (low, medium and high), marital status (married or other), employment status (no or yes, the retirement age for women in China was between 50 and 55 years old), area (Eastern, North-eastern, central), per capita annual family income (low, medium and high), history of smoking (yes or no), history of drinking (yes or no) and disease status (yes or no).

### Statistical Analysis

All analyses were conducted using Stata version 15.0 (Stata Corporation, College Station, TX, United States). The chi-square test was used for categorical variables and ANOVA for continuous variables. The preferences for the five food categories were recode “neutral,” dislike, and like. The multinomial logistic regression was used to assess the sociodemographic determinants of food preference. The multilevel mixed-effects linear regression was presented to assess the longitudinal association between food preference and PWB scores as all the participants attended the two surveys, with the predictors (food preference) were lagged. The PWB was estimated using data of 2011 and food preferences based on data of 2009. A negative regression coefficient indicates a decrease in PWB score. The following three models were used. Model 1 adjusted for preference for fast food, preference for salty snacks, preference for fruits, preference for vegetables, preference for sugary beverage and PWB scores from 2009; Model 2 further adjusted for age, educational level, area and per capita household income and Model 3 further adjusted for history of smoking, history of drinking and disease status. Urban–rural differences were analyzed through stratified analysis. Results are reported for relative risk ratio (RRR), beta coefficients (β), and 95% confidence interval (95% CI).

## Results

### Characteristics of the Study Sample

Table 1 presents the descriptive statistics for all outcome variables and covariates for the study sample. The average age of the respondents in this study is 48.8 years old, whilst the majority of participants were married (95.1% in 2009), still working (62.2% in 2009), not smoking (96.1%) and not drinking (89.6% in 2009). Most of the respondents were healthy without chronic nor acute diseases (85.3%). Educational level was not high in this sample with less than one third receiving high education. Participants from the rural area had lower educational level and per capita household income, tend to have a history of smoking and are likely to keep working (*p* < 0.001).

**TABLE 1:** Variables Used in Analysis of food Preference and Psychological well-being scores in the participants (*N* = 929).

Variables	Urban (*n* = 291)	Rural (*n* = 638)	Total (*n* = 929)	*p*-value
Score of psychological-well-being (2011)	9.71 (2.72)	9.91 (2.02)	9.85 (2.26)	0.21
**Independent variables (2009)**				
Preference of fast food				0.030*
Neutral	26(9.0%)	64(10.1%)	90(9.7%)	
Dislike	245(84.8%)	554(87.2%)	799(86.5%)	
Like	18(6.2%)	17(2.7%)	35(3.8%)	
Preference of salty snack				0.15
Neutral	43(14.9%)	89(14.0%)	132(14.3%)	
Dislike	214(74.0%)	499(78.6%)	713(77.2%)	
Like	32(11.1%)	47(7.4%)	79(8.5%)	
Preference of fruits				< 0.001***
Neutral	15(5.2%)	96(15.1%)	111(12.0%)	
Dislike	9(3.1%)	20(3.1%)	29(3.1%)	
Like	265(91.7%)	519(81.7%)	784(84.8%)	
Preference of vegetables				0.21
Neutral	18(6.2%)	53(8.3%)	71(7.7%)	
Dislike	6(2.1%)	6(0.9%)	12(1.3%)	
Like	265(91.7%)	576(90.7%)	841(91.0%)	
Preference of sweetened beverages				0.60
Neutral	74(25.6%)	167(26.3%)	241(26.1%)	
Dislike	157(54.3%)	358(56.4%)	515(55.7%)	
Like	58(20.1%)	110(17.3%)	168(18.2%)	
Demographics				
Age (Mean ± SD)	48.93 (2.74)	48.79 (2.70)	48.83 (2.71)	0.45
Education				< 0.001***
Low	62(21.3%)	291(45.8%)	353(38.1%)	
Medium	86(29.6%)	222(34.9%)	308(33.2%)	
High	143(49.1%)	123(19.3%)	266(28.7%)	
Marital status				0.053
Other	20(6.9%)	25(3.9%)	45(4.9%)	
Married	271(93.1%)	611(96.1%)	882(95.1%)	
Working status				< 0.001***
No	163(56.0%)	187(29.4%)	350(37.8%)	
Yes	128(44.0%)	449(70.6%)	577(62.2%)	
Area				0.23
Eastern	110(37.8%)	258(40.4%)	368(39.6%)	
North-eastern	71(24.4%)	175(27.4%)	246(26.5%)	
Central	110(37.8%)	205(32.1%)	315(33.9%)	
Income				< 0.001***
Low	57(20.0%)	192(30.4%)	249(27.2%)	
Medium	75(26.3%)	206(32.6%)	281(30.7%)	
High	153(53.7%)	233(36.9%)	386(42.1%)	
History of smoking				0.12
No	283(97.6%)	607(95.4%)	890(96.1%)	
Yes	7(2.4%)	29(4.6%)	36(3.9%)	
History of drink				< 0.001***
No	243(83.8%)	587(92.3%)	830(89.6%)	
Yes	47(16.2%)	49(7.7%)	96(10.4%)	
Disease of status				0.65
No	246(84.5%)	544(85.7%)	790(85.3%)	
Yes	45(15.5%)	91(14.3%)	136(14.7%)	

*p < 0.05; **p < 0.01; and ***p < 0.001.

Table 1 demonstrates that the average score of PWB in the whole sample was 9.85 in 2011. Most participants reported dislike of fast food (86.5% in 2009) and salty snacks (77.2% in 2009). More than 80% of the respondents like fruits, and more than 90% participants like vegetables. Nearly 20% participants liked soft beverages. No significant urban/rural difference was found in the preference for salty snacks, vegetables and sweetened beverages. Rural residents show less preference for fruits than urban respondents and rural residents were more likely to dislike fast food and (*p* < 0.05).

### Sociodemographic Determinants of Food Preference

After the adjustment for covariates, educational level, marital status, working status and history of smoking had no significant influence on participants’ food preference (*p* > 0.05). Table 2 showed that age only affected participants’ preference for fast food, with the older they got, the less they liked fast food. After adjusting for covariates, significant urban and rural discrepancy in food preferences was observed in our study. Participants from the rural areas, showed less preference for fast food, fruit and showed more preference for vegetables than urban participants. Participants from different geographic areas had different food preferences, those from North-eastern areas showed more dislike for salty snacks and sweetened beverages while those from central areas showed more like for salty snacks and fruit. Participants with high income showed more preferences for vegetables and fruits, while participants with history of drinking showed less dislike for sweetened beverages. There were significant differences in food preferences among people with the disease. They showed more dislike for fast food, vegetables, and sweetened beverages, while showed more like for salty snacks.

**TABLE 2:** Relative risk ratios (RRR) estimated by multinomial regression on associations between five food preferences and Sociodemographic factors.

Variables	Fast food		Salty snacks		Fruit		Vegetables		Sweetened beverages	
					
	Dislike	Like	Dislike	Like	Dislike	Like	Dislike	Like	Dislike	Like
**Age**	1.03	0.90*	1.04	0.94	1.08	0.99	1.01	0.99	1.02	0.94
**Region**										
Urban	1	1	1	1	1	1	1	1	1	1
rural	0.91	0.50*	0.95	0.73	0.75	0.68*	0.32*	0.96	1.12	1.03
**Education**										
Low	1	1	1	1	1	1	1	1	1	1
Medium	0.93	1.03	0.94	0.66	1.064	1.201	0.85	0.87	0.87	0.84
High	0.88	0.99	1.08	1.02	0.854	1.360	1.11	1.19	0.99	0.85
**Marital-status**										
Other	1	1	1	1	1	1	1	1	1	1
Married	1.61	1.30	1.46	1.56	1.413	1.240	0.28	0.78	1.26	1.19
**Working status**										
No	1		1	1	1	1	1	1	1	1
Yes	1.03	0.94	0.98	1.01	0.68	1.09	1.94	1.07	0.83	1.16
**Area**										
Eastern	1	1	1	1	1	1	1	1	1	1
North-eastern	1.18	1.13	1.93**	1.03	1.29	1.01	1.40	1.26	1.72**	1.36
Central	1.24	1.36	1.47	1.66*	1.25	1.79*	2.68	1.65	0.89	1.30
**Income**										
Low	1	1	1	1	1	1	1	1	1	1
Medium	0.99	1.05	0.84	0.84	0.94	0.95	1.12	1.26	0.82	0.73
High	0.88	1.57	0.76	0.93	1.179	1.60*	1.23	2.42**	0.98	0.95
**History of smoking**										
No	1	1	1	1	1	1	1	1	1	1
Yes	3.48	1.80	0.99	1.41	1.13	1.13	1.54	0.82	1.91	1.20
**History of drink**										
No	1	1	1	1	1	1	1	1	1	1
Yes	0.67	0.81	0.94	0.77	1.42	1.35	0.02	1.07	0.68*	1.32
**Disease of status**										
No	1	1	1	1	1	1	1	1	1	1
Yes	1.81*	1.23	1.92	2.45*	0.76	0.97	4.02**	1.15	1.43*	1.47

*p < 0.05; **p < 0.01. The neutral preference was treated as the reference category in the food preference options. The model was adjusted age, education level, area, per capita household income, history of smoking, history of drink and disease status.

### Association Between Food Preferences and PWB

Table 3 shows the multivariable adjusted association between PWB and different food preferences. PWB score was negatively associated with dislike for fruit, and positively with like sweetened beverages. Compared with participants who showed the neutral attitude for fruit and sweetened beverages, those who showed dislike for fruit had a lower PWB score while those who showed like for sweetened beverages may had higher PWB score. In fully adjusted models (model 3), those who those who showed the dislike for fruits and those who showed the like for sweetened beverages had regression (95% CI) for the PWB score of −1.26, (−2.21–0.321) and 0.66 (0.20–1.11), respectively. The same results were found only among participants in urban areas, with beta coefficient ranging from −2.61(95% CI = −4.83, −0.39) for dislike fruit to 1.02(95% CI = 0.09, 1.95) for like sweetened beverages. Besides, the PWB score of rural participants was negatively associated with the preference for snacks in fully adjusted model, with beta coefficient −0.83 (95% CI = −1.58, −0.09), while this association is flipped (though not significant) in the urban population. Additionally, we found the PWB score was not significantly associated with preference for fast food, salty snacks and vegetables in the whole samples (*p* > 0.05).

**TABLE 3:** Association (beta coefficient) between food preference and PWB score by multivariable mixed linear regression among perimenopause period women.

Variables	Model 1			Model 2			Model 3		
			
	All	Rural	Urban	All	Rural	Urban	All	Rural	Urban
**Preference for fast food (lagged in 2009)**									
Dislike	0.07 (−0.51,0.66)	0.13 (−0.49,0.74)	−0.14 (−1.48,1.20)	0.14 (−0.45,0.72)	0.19 (−0.40,0.78)	−0.07 (−1.47,1.33)	0.16 (−0.43, 0.74)	0.23 (−0.36,0.82)	−0.12 (−1.50,1.26)
Like	0.24 (−0.73,1.21)	−0.39 (−1.53,0.76)	0.21 (−1.64,2.05)	0.18 (−0.79,1.14)	−0.67 (−1.77,0.44)	0.45 (−1.48, 2.37)	0.17 (−0.79, 1.13)	−0.61 (−1.71,0.50)	0.63 (−1.28,2.53)
**Preference for salty snacks (lagged in 2009)**									
Dislike	−0.17 (−0.67,0.32)	−0.11 (−0.64,0.42)	−0.38 (−1.47,0.71)	−0.29 (−0.78,0.21)	−0.32 (−0.84,0.20)	−0.34 (−1.47,0.78)	−0.30 (−0.79,0.20)	−0.36 (−0.87,0.16)	−0.24 (−1.36,0.88)
Like	−0.25 (−0.94,0.44)	−0.90* (−1.66, −0.14)	0.67 (−0.73,2.07)	−0.19 (−0.88,0.50)	−0.82* (−1.57, −0.08)	0.61 (−0.84,2.06)	−0.20 (−0.89,0.49)	−0.83* (−1.58, −0.09)	0.51 (−0.92,1.94)
**Preference for fruits (lagged in 2009)**									
Dislike	−1.22* (−2.18, −0.26)	−0.13 (−1.14,0.89)	−3.35** (−5.59, −1.10)	−1.31** (−2.26, −0.36)	−0.21 (−1.20,0.78)	−2.87* (−5.12, −0.62)	−1.26** (−2.21, −0.31)	−0.31 (−1.30,0.68)	−2.61* (−4.83,−0.39)
Like	−0.44 (−0.95,0.07)	−0.30 (−0.80,0.20)	−0.43 (−1.97,1.11)	−0.41 (−0.92,0.10)	−0.31 (−0.810.18)	−0.06 (−1.62,1.50)	−0.39 (−0.90,0.18)	−0.29 (−0.78,0.21)	−0.22 (−1.76,1.32)
**Preference for vegetables (lagged in 2009)**									
Dislike	0.39 (−1.06,1.84)	−0.29 (−2.08,1.50)	1.57 (−0.97,4.11)	0.39 (−1.04,1.82)	0.10 (−1.63,1.83)	1.21 (−1.39,3.80)	0.42 (−1.01, 1.85)	0.15 (−1.58,1.877)	1.47 (−1.09,4.03)
Like	0.60 (−0.02,1.23)	0.38 (−0.28,1.03)	0.98 (−0.44,2.41)	0.47 (−0.15,1.09)	0.38 (−0.26,1.01)	0.70 (−0.77,2.16)	0.47 (−0.15, 1.09)	0.36 (−0.27,1.00)	0.94 (−0.51,2.39)
**Preference for sweetened beverages (lagged in 2009)**									
Dislike	0.33 (−0.04,0.69)	0.23 (−0.16,0.62)	0.58 (−0.20,1.35)	0.27 (−0.09,0.64)	0.13 (−0.25,0.52)	0.56 (−0.21,1.34)	0.25 (−0.11, 0.62)	0.11 (−0.27,0.49)	0.52 (−0.25,1.29)
Like	0.69** (0.24,1.15)	0.54* (0.04,1.03)	0.90 (−0.04,1.83)	0.67** (0.21,1.12)	0.45 (−0.04,0.94)	1.03* (0.09,1.98)	0.66** (0.20,1.11)	0.45 (−0.04,0.93)	1.02* (0.09,1.95)

*p < 0.05; **p < 0.01. The neutral preference was treated as the reference category in the food preference options. The five food preference choices were lagged in 2009. Model 1 was adjusted for preference of fast food, preference of salty snacks, preference of fruits, preference of vegetables, preference of sugary beverage. Model 2 was adjusted the same as Model 1 plus age, education level, area, per capita household income. Model 3 was adjusted the same as Model 2 plus history of smoking, history of drink and disease status.

## Discussion

Around the world, about 10,000 women enter perimenopause period each year (Landis and Moe, 2004). Perimenopausal syndrome is not only a physiological change in perimenopausal women, but also psychological changes, which affect each other and aggravate each other. These changes may also have an impact on food intake and food preference of women in premenopausal period. This study focuses on the association on preference and PWB of Chinese women in perimenopausal period. Most women in perimenopausal period preferred vegetables and fruits than fast food and salty snacks. Moreover, nearly 20% of women reported that they like sweetened beverages in the longitudinal survey. We found the participants with different age, income, disease of status and different region and areas have different food preference. Accordingly, we controlled these confounding variables in multilevel mixed-effects linear regression analysis.

In China, fast food and salty snacks were often called Western fast food, and the concept was not novel to respondents. In our study, preference for Western fast food (fast food and salty snack) was not always associated with poor psychological health status. This finding is different from previous studies in adolescents or adults, which showed fast food consumption be associated with poorer psychological condition (Sclafani, 2001; Sanchez-Villegas et al., 2012; Zahedi et al., 2014). As the rapid change of dietary in China, food restaurants have proliferated and with more processed and convenient. The number of fast food restaurants increased from 929,125 to 1,981,019 between 2004 and 2012 (Wang et al., 2016). Although this study did not find significant difference between fast food and PWB, the relationship of fast food consumption with PWB of Chinese women in perimenopausal period should be studied further.

The findings presented that women in perimenopausal period who showed dislike for fruits were less likely to report higher PWB score. The previous studies pointed out that consumption of fresh vegetables and fruits associated with PWB, and which could increase happiness, life satisfaction (Mujcic and Oswald, 2016; Sapranaviciute-Zabazlajeva et al., 2017). However, our results regarding preferences for vegetables is not found a significant association with the PWB score, which is similar to the results of another cohort study in Australia (Mihrshahi et al., 2015). As previous research reported that consumption of fruit and vegetable may be related to reduced risk of both all-cause mortality and cardiovascular-related mortality and individuals with higher PWB may self-report healthier behaviors (Boehm et al., 2018). More research about the PWB and the preference of vegetables and fruit and time of follow-up are needed.

The paradoxical finding of this study is that PWB score was positively associated with the preference of sweetened beverages, while the previous studies found that high consumption of sugar-containing soft drinks were associated with mental health problems among adolescents (Lien et al., 2006; Estherlydia and John, 2009; Shi et al., 2010). The differences between our result and previous studies could be explained to the special population women in the premenopausal period, who may have insufficient progesterone or estragon content which reducing the secretion of pleasant neurotransmitters (serotonin, dopamine and norepinephrine) Volume. A longitudinal study showed that perimenopausal women were 2.5 times more likely to be depressed than non-perimenopausal women (Easterlin, 2003). Women in perimenopausal period may have craving for sweet taste, which may contribute to serotonergic, dopaminergic secretion through different physiological mechanisms (Kim et al., 2017). Moreover, individuals who prefer sweetened beverages might be unaware of the harmful on physical health. Nevertheless, consumption of sweetened beverages is of public health concern in China. Not only does consumption of sweetened beverages is associated with increased risk of hypertension (Kim and Je, 2016; Kwak et al., 2019), cardiovascular disease (Mekonnen et al., 2013), obesity (Grimes et al., 2013), and diabetes (Liese et al., 2015), sweetened beverages intake is also strongly associated with endometrial cancer. Since no other sweet foods were studied in this study, this result may need further verification.

We found that the perimenopausal women in urban and rural areas have different food preferences. Compared with urban perimenopausal women, rural perimenopausal women show less like for fast food and fruit and more dislike for vegetables. The stronger association between PWB score and the preference for fruit was founded in urban participants than the whole sample. And the PWB score of urban participants was positively associated with the preference of sweetened beverages. The consumption of fruits and vegetables in urban areas is higher than that in rural areas in recent years in China (He et al., 2016), and consumption rate of sweetened beverages in urban areas is higher (Li et al., 2014) may be the main reason. Further research on the differences between urban and rural areas, whether it is dietary preferences or mental health were necessary.

The results from the present study was interpreted with caution as there are some limitations. Firstly, we don’t have access to the FFQ data collected only in 2015. Therefore, we only used the food preferences data to determine dietary practice. In a way, the food preference can indicate both individuals’ food consumption habits and their attitudes for different types of food for long-term than 3-day 24 h dietary recall method (Lee et al., 2018). More various or objective indicators of food consumption should be used in future studies. Secondly, the use of self-reported data can potentially introduce recall bias, especially for variables based on participants’ long-term memory. Self-reporting is also subject to dishonest responses or acquiescence bias. Another limitation of the study is the use of a three item PWB, not the comprehensive tools such Pleasure (CASP-19) scale. Thirdly, the definition of perimenopause period in this study is mainly focused on age, and the subjective description of perimenopausal symptoms should be added to measure the results of this population. Further research is necessary with comprehensive related questions. Fourthly, the time of follow-up in this study is short which only provide the limited evidence. Further study to investigate the PWB and food preference and more temporal follow-up are needed. Finally, Chinese women whose food preference and social role may be unique to the Chinese and it might limit the generalizability. Despite these limitations, this study is unique in pointing toward a potential causal linkage between food preference and PWB. This study is one of the few scientific attempts to examine the association between food preferences and PWB among perimenopausal women in China. Further research is required to establish causality. Additionally, the longitudinal data provided stronger evidence than the cross-sectional study in China.

## Conclusion

Psychological well-being score was negatively associated with the dislike for fruit but positively associated with the preference for sweetened beverages, especially among participants from urban areas. Women in perimenopausal period who liked fast food, salty snacks may not always be associated with poor psychological health status after controlling the confounding variables. Improving the PWB of perimenopausal women may be possible with increasing in the consumption of fruit, especially among those living in urban areas. Given the adverse effects of sweetened beverages, more research was need between PWB and the sweetened beverages, which should not be promoted.

## Data Availability Statement

The datasets generated for this study are available on request to the corresponding author.

## Ethics Statement

The studies involving human participants were reviewed and approved by the institutional review committees of the University of North Carolina (United States) and the National Institute of Nutrition and Food Safety (China). The patients/participants provided their written informed consent to participate in this study.

## Author Contributions

TW conducted the statistical analyses of the data and prepared the manuscript. YZ and FZ helped to revise the manuscript. XH, MS, and ZS helped to review and provide critical comments to the manuscript. All authors checked and proofread the final version of the manuscript.

## Conflict of Interest

The authors declare that the research was conducted in the absence of any commercial or financial relationships that could be construed as a potential conflict of interest.
